# FEEDBACK trial - A randomised control trial to investigate the effect of personalised feedback and financial incentives on reducing the incidence of road crashes

**DOI:** 10.1186/s12889-023-16886-z

**Published:** 2023-10-18

**Authors:** Mark Stevenson, Duncan Mortimer, Lynn Meuleners, Anthony Harris, Teresa Senserrick, Jason Thompson, Anurika De Silva, Humberto Barrera-Jimenez, Avita Streatfield, Maneesha Perera

**Affiliations:** 1 Transport, Health and Urban Systems Research Lab, Melbourne School of Design, University of Melbourne, Melbourne, Australia; 2 Faculty of Engineering and IT, University of Melbourne, Melbourne, Australia; 3 Centre for Health Economics, Monash University, Clayton, Australia; 4 Western Australian Centre for Road Safety Research, School of Psychological Science, University of Western Australia, Perth, Australia; 5 Centre for Epidemiology and Biostatistics, Melbourne School of Population and Global Health, University of Melbourne, Melbourne, Australia; 6 Methods and Implementation Support for Clinical and Health (MISCH) Research Hub, Faculty of Medicine, Dentistry and Health Sciences, University of Melbourne, Melbourne, Australia

**Keywords:** Road injury, Driver feedback, Injury prevention, Road crashes, Smart financial incentives

## Abstract

**Background:**

Road crashes continue to pose a significant threat to global health. Young drivers aged between 18 and 25 are over-represented in road injury and fatality statistics, especially the first six months after obtaining their license. This study is the first multi-centre two-arm parallel-group individually randomised controlled trial (the FEEDBACK Trial) that will examine whether the delivery of personalised driver feedback plus financial incentives is superior to no feedback and no financial incentives in reducing motor vehicle crashes among young drivers (18 to 20 years) during the first year of provisional licensing.

**Methods:**

A total of 3,610 young drivers on their provisional licence (P1, the first-year provisional licensing) will participate in the trial over 28 weeks, including a 4-week baseline, 20-week intervention and 4-week post-intervention period. The primary outcome of the study will be police-reported crashes over the 20-week intervention period and the 4-week post-intervention period. Secondary outcomes include driving behaviours such as speeding and harsh braking that contribute to road crashes, which will be attained weekly from mobile telematics delivered to a smartphone app.

**Discussion:**

Assuming a positive finding associated with personalised driver feedback and financial incentives in reducing road crashes among young drivers, the study will provide important evidence to support policymakers in introducing the intervention(s) as a key strategy to mitigate the risks associated with the burden of road injury among this vulnerable population.

**Trial registration:**

Registered under the Australian New Zealand Clinical Trials Registry (ANZCTR) - ACTRN12623000387628p on April 17, 2023.

**Supplementary Information:**

The online version contains supplementary material available at 10.1186/s12889-023-16886-z.

## Background

Road crashes are predicted to be the fifth leading cause of death worldwide by 2030 [1]. Despite early successes in reducing the burden of road injury, road crashes remain a leading cause of mortality and morbidity in Australia [2]. Over the past decade, there have been no significant declines in the rate of road deaths and serious injury [2]. Consequently, an urgent rethink of road trauma prevention measures is needed if Australia is to achieve its national road safety targets of reducing fatalities by 50% and serious injuries by 30% by 2030 [3].

Approaches to reducing road deaths and serious injury must account for the overrepresentation of young drivers in road crashes. Road injury is one of the leading causes of death among young Australians, accounting for 1 in 5 deaths among people aged 15–24 [4, 5]. Despite only comprising 14% of all licence-holders, young drivers are involved in one-quarter of all serious crashes and remain over-represented in crash statistics, with the risk of a crash being greatest in the first year of a young driver’s licensure [5, 6].

The FEEDBACK trial builds on past research indicating that feedback and financial incentives can reduce risky driving behaviours [7, 8]. Some studies found that personalised safety feedback to drivers led to improved driving behaviours, like reducing speeding and harsh braking [9–12]. Others suggest that feedback alone may be insufficient to motivate behaviour change, and that feedback should be combined with financial incentives in order to deliver significant reductions in risky driving [8, 13–15]. Stevenson et al. [8], for example, found that the effects of feedback alone did not yield statistically significant reductions in risky driving behaviours, whereas feedback combined with low-cost financial incentives had a significant effect.

Similarly, providing financial incentives cannot be assumed to improve driving performance. Although there is evidence that financial incentives can motivate behaviour change – like smoking cessation, physical activity, vaccination, and screening [16, 17] – the effects of such incentives do not always generate the intended outcomes [7, 18, 19]. Instead, incentives can create unintended consequences whilst doing little to influence the behaviour of already-safe drivers [7, 20, 21]. For instance, Mortimer et al.’s [7] study found that providing and withdrawing an incentive completely offset the gains in driving performance while the incentive was ‘switched-on’, leaving affected drivers worse off than they started. These studies underscore the instrumental role of careful incentive design.

The FEEDBACK trial will use ‘smart incentives’: low-cost, loss-framed incentives that are personalised based on the drivers’ behaviour. Studies aiming to reduce traffic congestion [22–24], improve drivers’ fuel efficiency [25], and modify commuters’ travel behaviours [26, 27] all highlight the utility of offering personalised incentives – rather than static rewards to trigger behaviour change. Whereas previous studies have provided penalties (or rewards) whenever the incentivised behaviour fell beneath a *fixed* threshold [8], thresholds for incentives in the FEEDBACK trial will be driver-specific and set to require (achievable) improvement relative to previous driving behaviour. These driver-specific thresholds will be progressively lowered – month on month – to incentivise further improvements, even among safer drivers.

Incentives in the FEEDBACK trial will leverage loss-aversion by imposing monthly penalties for risky driving, rather than rewards for safe driving [28]. Penalties will be framed as deductions from an upfront payment to address differences in acceptability between rewards and penalties. This approach mimics a Pay-As-You-Drive (PAYD) implementation, where financial penalties are typically implemented as periodic deductions from an earned discount on PAYD insurance premiums [29, 30].

Whilst previous research indicates that feedback and incentives can improve driving behaviours [7, 8, 28], this study will also evaluate the likely impact of personalised feedback and incentives on crash risk specifically. Moreover, this trial marks the first time that such an intervention will be evaluated among young drivers in their first year of licensing in relation to crash outcome. This evaluation will provide policymakers with crucial evidence on the effects of population-scale interventions, such as insurance premiums being linked to safer driver behaviours.

## Methods and design

### Overview of the study design

The FEEDBACK trial is a two-arm parallel group, superiority randomised controlled trial. The study was approved by the Human Research Ethics Committee of the University of Melbourne (HREC 023-25244-42050-4), the Queensland University of Technology Research Governance and Integrity team (Project ID: 7478) and the University of Western Australia (2023/ET000616). It was funded by the National Health and Medical Research Council Australia Partnership Grant (NHMRC; Grant number: 2,015,470). The trial was prospectively registered with Australian New Zealand Clinical Trials Registry (ANZCTR; ACTRN12623000387628p).

The trial was designed to comply with Standard Protocol Items: Recommendations for Interventional Trials (SPIRIT) [31] and Good Clinical Practice guidelines. Trial findings will be reported in accordance with the Template for Interventional Description and Replication (TIDier) checklist [32] and Consolidated Standards of Reporting Trials (CONSORT) guidelines [33].

Following NHMRC guidelines, a trial Data Safety and Monitoring Committee (DSMC) will be established. The DSMC will be a multidisciplinary group who will have no involvement in the trial nor any conflicts of interest. It will include individuals with scientific expertise in transport safety research, statistics, and a consumer representative to ensure end-user representation and engagement during the trial. The committee will convene once per year to monitor the efficacy and safety of the trial. The responsibility of the committee will be to consider reasons for ineligibility and non-participation, adherence to protocol, intervention fidelity, study events, adverse events (those perceived to be related to the devices such as smartphones), withdrawals and deaths, and provide recommendations to the chief investigator about continuing, modifying, or stopping the trial. There are no planned interim analyses or stopping guidelines.

#### Aims and eligibility criteria

The FEEDBACK trial will examine whether personalised driver feedback and personalised financial incentives are superior to *no* personalised driver feedback and incentives in reducing motor vehicle crashes among drivers aged 18–20. To participate in the trial, drivers are required to meet the following eligibility criteria. Additionally, they will need to provide informed consent and complete an online survey that includes questions on demographic characteristics, their driving experience, and attitudes towards driving.

- Aged 18 to 20 years when joining the trial.
- Live in one of three Australian states: Queensland (QLD), New South Wales (NSW), or Western Australia (WA).
- Hold a provisional (P1) licence for a car.
- Have access to a vehicle with Bluetooth functionality that they drive regularly.
- Have a smartphone that is an Android or an iPhone 6 or newer.

When consent has been provided, participants will receive a link to download a smartphone application (‘FEEDBACK’ app), developed by industry partner Urban Analytica (UA) Pty Ltd and will be asked to pair the smartphone with their car via Bluetooth. Each participant’s driving behaviour will be monitored for 28 weeks, beginning with a 4-week baseline period.

After the 4-week baseline period, each participant will be randomised into one of two groups: (1) an intervention group that will receive personalised driver feedback and personalised financial incentive, or (2) a control group that *does not* receive personalised feedback or financial incentive. The intervention period runs for 20-weeks. The trial will conclude with a further 4-week post-intervention period. Participants will receive no feedback nor incentives during the post-intervention period but will be monitored to evaluate the persistence of the hypothesised treatment effect after the intervention ceases. The trial will conclude by asking participants to complete an online questionnaire.

In order to keep participants engaged throughout the trial, both the intervention and control groups will be entered into a weekly prize draw during the 20-week intervention period and at the end of the trial. Engagement in the trial will involve keeping location tracking for the FEEDBACK app enabled throughout the trial so that the app can collect driving data.

### Delivery of the intervention via mobile telematics

Vehicle telematics refers to an integrated system in which telecommunications are used to transmit information captured by sensors to record real-time data about a vehicle’s operations, such as speed, acceleration, deceleration, and GPS location [34]. Applications of telematics are diverse, encompassing vehicle fleet management, vehicle diagnostics, insurance, and usage-based services [34, 35]. Furthermore, the role of telematics is gaining traction as a strategy for providing personalised feedback to drivers about crash risk indicators, which include speeding (i.e., travelling above posted speed limits) and the frequency of harsh braking and acceleration [8, 36].

While traditional telematics data collection relies on costly in-vehicle sensors, advancements in smartphone technology have enabled the capture of telematics data using low-cost smartphone sensors [37]. Furthermore, as most young drivers own a smartphone, smartphone-based telematics - hereafter referred to as ‘mobile telematics’ - presents a promising and affordable alternative for collecting telematics data from a larger population [34, 35]. Mobile-telematics technology will enable delivery of both personalised feedback and financial incentives in the FEEDBACK intervention by capturing real-time data on adverse driving behaviours that contribute to crash-related injuries such as speeding, harsh braking, and harsh acceleration.

### Study timeline

Each participant will be involved in the trial for a total of 28 weeks, consisting of a 4-week baseline period, 20-week post-randomisation (intervention) period, and 4-week post-intervention period. To join the trial, a participant will sign a consent form and complete a pre-trial questionnaire. Upon consent and completion of the pre-trail questionnaire participants will receive a link via SMS to download the FEEDBACK app. Once a participant downloads the FEEDBACK app and pairs the smartphone with their car (via Bluetooth), their participation in the trial will commence.

**Fig. 1:**
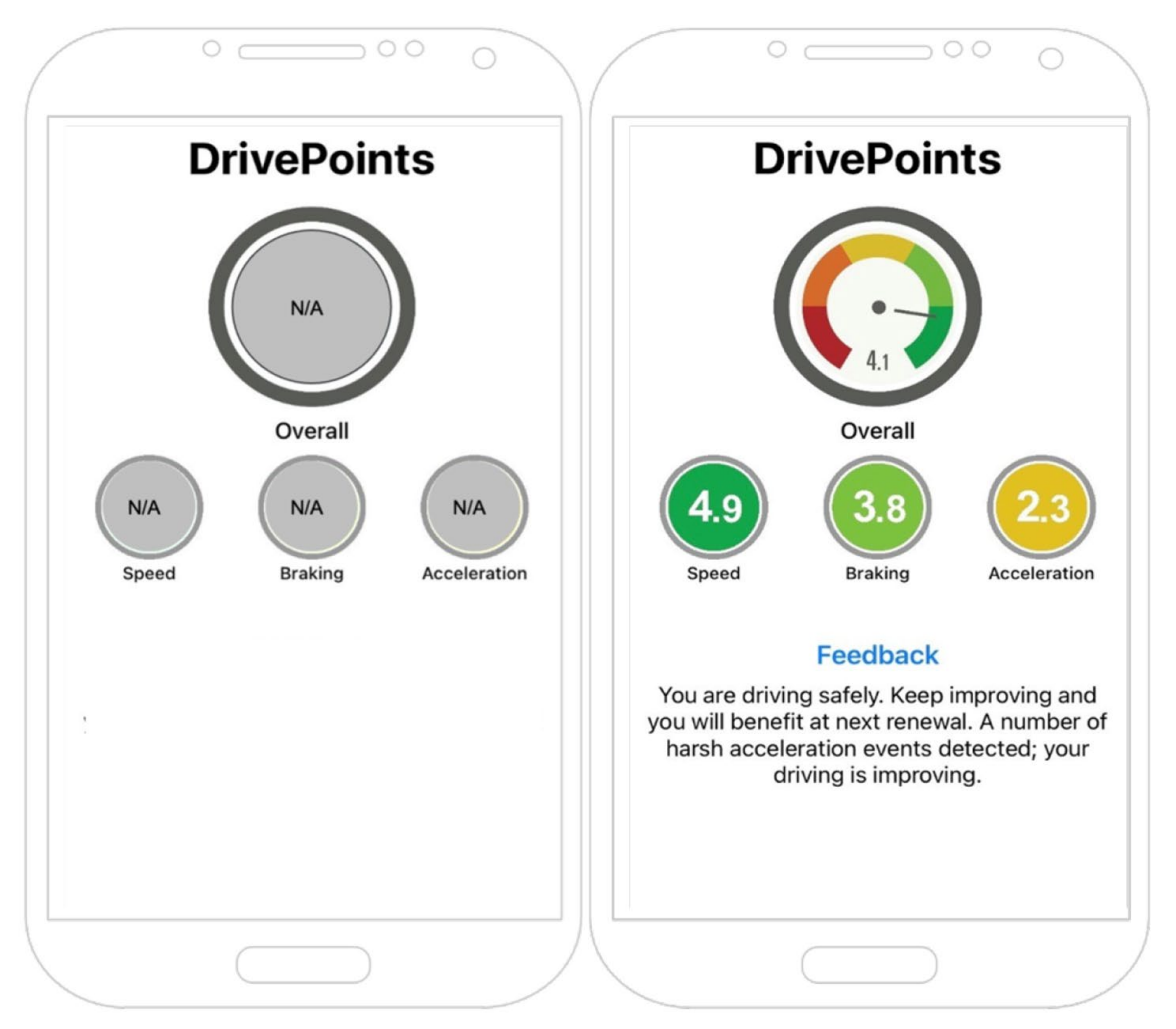
An illustration of the FEEDBACK app. (**a**) FEEDBACK app view during the baseline and post-intervention periods for both groups and during the intervention period for the control group. (**b**) FEEDBACK app showing the ‘DrivePoints’ scores and feedback for the intervention group during the intervention period

During the 4-week baseline period, the FEEDBACK app will collect data on the participant’s driving behaviours: speeding, braking, and acceleration. However, the app will not display any information to the participants during the baseline period (see Fig. 1a). After the 4-week baseline, participants will be randomised (as discussed in Sect. 2.6) into either the control group or intervention group for the next 20-week intervention period.

#### Control group

During the 20-week intervention period, participants randomised to the control group will be entered into a weekly draw to win an e-voucher worth AU$100 and will receive weekly SMS messages announcing the winner of the weekly draw. Weekly SMS messages will also include a reminder to keep location tracking enabled for the FEEDBACK app. Throughout the trial, the FEEDBACK app will not display any information to the control group participants as shown in Fig. 1a.

#### Intervention group

The intervention group will be entered into the same weekly draw and in addition, will receive personalised driver feedback and financial incentives as described below.

*Personalised driver feedback*: Participants in the intervention group will be able to view feedback on their driving performance in the FEEDBACK app. This feedback is provided in the form of a ‘DrivePoints’ score, which is an average of scores calculated for speed, braking, and acceleration captured via mobile telematics for driving trips over a week. The DrivePoints score ranges in value between 0 and 5 and is colour coded as dark green, light green, yellow, amber and red, where dark green indicates the lowest level of at-risk driving, light green, yellow and amber indicate increasing levels of at-risk driving, and red indicates the highest level of at-risk driving. For example, for weeks with repeated instances of speeding, harsh braking or acceleration, the DrivePoints score will be classified as yellow, amber or red, depending upon the frequency and severity of each behaviour. For weeks with low or no unsafe driving, the DrivePoints score will be classified as light green or dark green. Figure 1b shows an example of how the DrivePoints scores will be visible to the participants through the FEEDBACK app.

Each month the intervention group will receive an SMS containing their DrivePoints score for that month and a target score (referred to herein as the ‘safe driving target’) for the following month. Safe driving targets are calculated per participant and based on their driving behaviour in the month prior as indicated by the number of red, yellow, amber, light green and dark green DrivePoint scores. For instance, if a driver receives mostly green DrivePoints scores and no red scores but some yellow and at least one amber DrivePoint score in a given month, then their safe driving target in the following month would be to maintain their record of mostly green and no red DrivePoint scores but to also avoid getting any amber scores and to receive no more than one yellow score. The ‘safe-driving target’ for the first month (Month 1) will be personalised using the data from the baseline period. For every other month, the safe-driving target will be calculated based on driving behaviour in the month prior. Table 1 defines rules for calculating safe driving targets.

**Table 1:** Calculations of the safe driving target for each month during the intervention period^1^

Driving performance in the previous month	Safe-driving target for the current month
No trips	No more than one yellow, amber or red DrivePoint scores, *or* repeat of previous month’s safe-driving target, whichever is the most stringent.
Only dark green DrivePoint scores	Only dark green DrivePoint scores
Only dark green and light green DrivePoint scores	Only dark green DrivePoint scores
At least one yellow, but all other DrivePoint scores were light green or dark green	Only dark green and light green DrivePoint scores with no more than one light green
Any number of yellow, light green and dark green DrivePoint scores, at least one amber DrivePoint score, and no red DrivePoint scores	Only yellow, light green and dark green DrivePoint scores, with no more than one yellow
Any number of amber, yellow, light green and dark green DrivePoint scores, with one red DrivePoint score	Only amber, yellow, light green and dark green DrivePoint scores, with no more than one amber
Any number of amber, yellow, light green and dark green DrivePoint scores, with two red DrivePoint scores	Any number of amber, yellow, light green and dark green DrivePoint scores, with only one red DrivePoint score
Any number of amber, yellow, light green and dark green DrivePoint scores, with at least three red DrivePoint scores	Any number of amber, yellow, light green and dark green DrivePoint scores, with no more than two red DrivePoint scores

^1^ Decision rules specified in Table 1 will be applied in each month of the intervention period subject to two constraints. First, the ‘safe-driving target’ for a given month could not be less stringent than the participant’s ‘safe-driving target’ in any prior month. Second, the floor for acceptable driving behaviour could only be specified in the first month of the intervention period to ensure that penalties were applied for high and persistent levels of unsafe driving. The application of these constraints and the effects of the incentive should result in a progressive lowering of the threshold over the 20-week intervention period to incentivise further improvements in driving behaviour

#### Personalised financial incentives

Financial incentives will be structured as penalties for risky driving; levied as monthly deductions from an upfront payment of AU$120 deposited to a virtual ‘safe driving account’. This approach is designed to mimic a PAYD implementation, where financial penalties are typically framed as periodic deductions from an (earned) discount on PAYD insurance premiums. If a participant fails to meet their safe driving target for a given month, AU$24 will be deducted from their safe driving account that month. Participants will receive no penalty for risky driving provided that their driving behaviour remains consistent with achievement of their safe driving target (e.g., yellow and amber DriveScores would not incur a penalty if the monthly safe driving target is no more than one red DriveScore). Each week, the intervention group participants will receive an SMS updating them on the weekly draw, their current DrivePoints score and safe driving target, and safe driving account balance (see Table 2).

**Table 2:** An example SMS message a participant in the intervention group receives during the intervention period

Thank you for completing another month in the FEEDBACK trial. Your safe driving account currently has a balance of $**120**, and your new safe driving target is: **only yellow, light, or dark green DrivePoints scores, with no more than ONE yellow** over the next four weeks. If you fall short of this safe-driving target, you will lose $24 from your safe-driving account.	

After the 20-week intervention period, there will be a 4-week post-intervention period. During this time, participants will not be entered into weekly draws. The intervention group will also not be able to view information in the FEEDBACK app, nor receive SMS messages with updates on their DrivePoints scores, safe driving targets, or safe driving account balance. However, they will receive an SMS message reminding them to keep location tracking enabled for the FEEDBACK app. Mortimer et al.’s [7] study illustrates how a target behaviour may decay or reverse once an intervention concludes, with findings showing behavioural reversals once feedback and incentives are ‘switched off’. Monitoring participants’ driving behaviour during this post-intervention period will reveal whether any changes in participants’ driving performance persist after the intervention ceases.

**Fig. 2:**
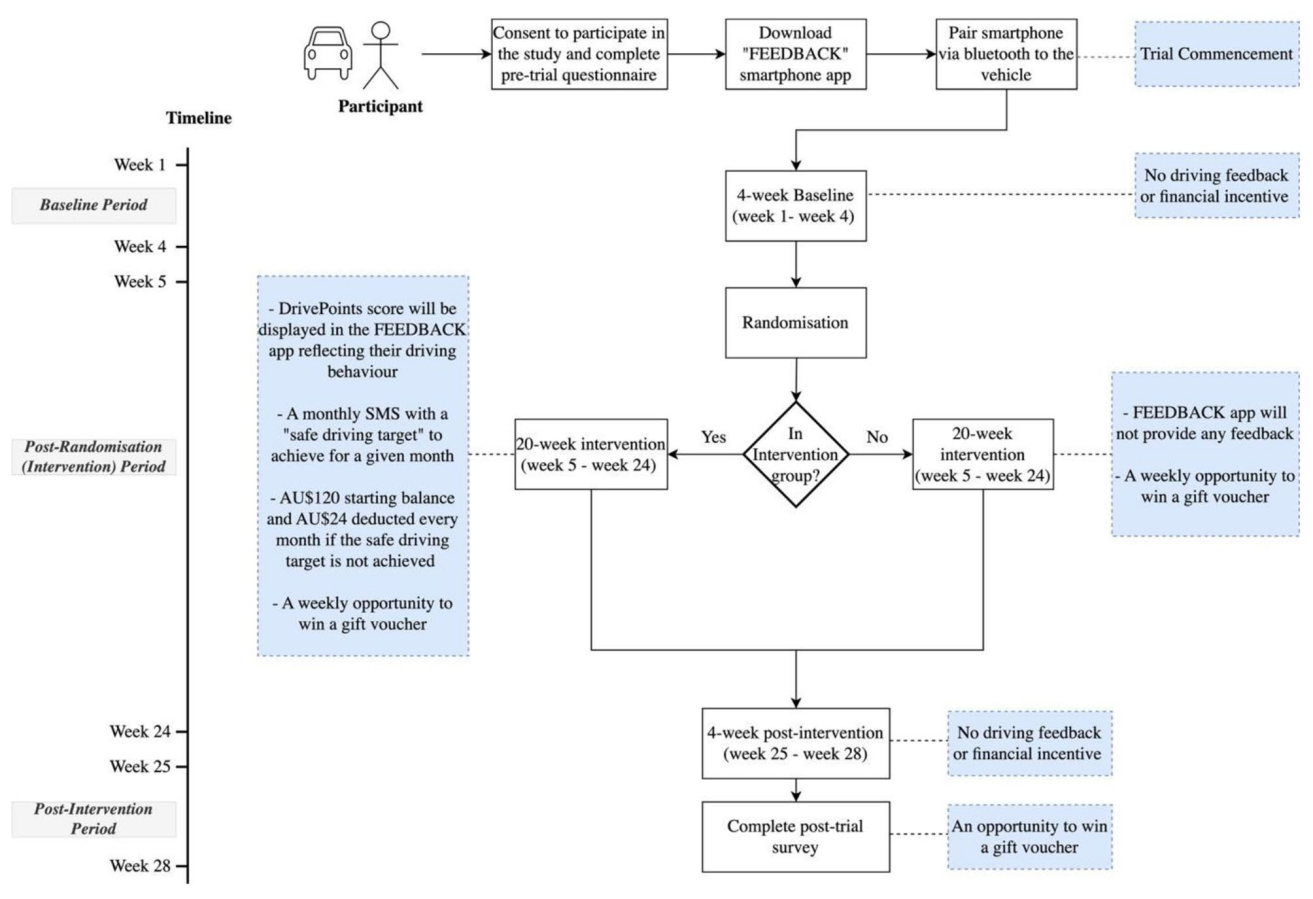
An overview of the FEEDBACK Trial illustrating the activities during each stage of the trial: 4-week baseline, 20-week post-randomisation (intervention) and 4-week post-intervention period

At the end of this post-intervention period, participants will receive a link to the post-trial questionnaire. Upon completing the questionnaire, the control group will be entered into one final draw. The intervention group will be able to receive the remaining funds of their safe driving accounts via an e-voucher. Figure 2 illustrates an overview of the aforementioned activities conducted during each stage of the trial timeline.

Participants can withdraw from the trial at any time but will not receive financial compensation thereafter. To withdraw, a participant can contact one of the trial coordinators via email. If a participant does not inform the trial coordinators of their withdrawal but the research team no longer receives telematics data from a participant, a research staff member will call the participant to confirm whether the participant has removed the FEEDBACK app or disabled location services and whether they still intend to participate in the trial.

### Hypotheses and outcome measures

The **primary hypothesis** will assess whether personalised safety feedback and personalised financial incentives reduce the incidence of police-reported crashes of any type during the 24-week intervention and post-intervention periods. The primary outcome is a police-reported crash over the 24-week intervention and post-intervention periods. The research team will obtain data on police-reported crashes using drivers’ licence numbers provided by participants in the pre-trial questionnaire.

The **secondary hypotheses** will assess the effects of personalised feedback and financial incentives on:

1. Reducing the incidence of self-reported crashes by young drivers.
2. Improving drivers’ safety scores (‘DrivePoints’ scores) from baseline.
3. Reducing crash risk behaviours identified by speeding, harsh braking, hard acceleration, and drowsy driving from baseline.

The **outcomes** to assess the secondary hypotheses are:

1. Self-reported involvement in a crash that took place during the 24-week intervention and post-intervention periods that resulted in damage to their own or someone else’s vehicle.
2. DrivePoints score at baseline and at weekly intervals through to week 28.
3. Driving behaviours, namely:
  Speeding (defined as 10 km/h over the speed limit).
  Harsh braking (sudden braking in which items in the vehicle would move around, defined in telematics technology as a -0.5 g force).
  Hard acceleration (rapid acceleration exceeding a 0.25 g force).

### Sample size

Statistics from QLD and NSW in Australia in 2022 suggest there is a large population of P1 licence holders aged 18–20 years to sample from: 32,151 young drivers in QLD [38] and 42,039 in NSW [39]. Based on crash statistics for QLD from 2016 to 2022, the incidence of a young P1 licence holder having a police-reported crash of any type during a 6-month period is approximately 3% [40], with similar patterns expected across NSW and WA. Based on the findings of recent research [7, 8] the FEEDBACK trial intervention is expected to lower the proportion of a police-reported crashes from 3 to 1.5%.

To detect a 1.5% absolute reduction in the incidence of young drivers having a police-reported crash of any type during the 24-week intervention and post-intervention periods, from 3% in the control group to 1.5% in the intervention group (or 0.5 relative risk), using a two-sided type I error of 5% and a power of 80%, a total of 3,068 participants are required. Allowing for a 15% non-response rate due to drivers who may choose to withdraw from the study, turn off their phones, or delete the smartphone app, 1,805 participants per arm, or a total of 3,610 participants, will be recruited to participate in the trial.

To achieve adequate participant enrolment and reach the target sample size, recruitment strategies will be carried out in partnership with government stakeholders. The transport departments from the three states of Western Australia, Queensland, and New South Wales will assist with advertising the trial via several media channels, including social media, associated websites, and advertising boards in licencing centres.

### Randomisation and blinding

Following the 4-week baseline period, eligible drivers will be randomised to intervention or control using randomly permuted blocks of varying sizes in a 1:1 ratio, stratified by study site (state) and sex (male/female). The randomisation schedule will be computer-generated by an independent statistician and stored on a password-protected database at the University of Melbourne, managed by a researcher who is not one of the investigators. It will not be possible to blind participants due to the nature of the intervention. However, chief investigators will be blinded to group allocation during the statistical analysis. Main statistical analyses will be performed blinded to group details.

## Data and analysis

### Data collection

#### Pre-trial questionnaire

The pre-trial questionnaire will be disseminated online via Qualtrics, an online survey platform. It is composed of an introductory section, including copies of the plain language statement and informed consent form. Upon consent, participants will be asked to enter their driver’s licence number, mobile number, and email address. The questionnaire includes questions regarding demographics including [specify demographics], driving experience and attitudes to risky driving. Attitudes to risky driving questions were adapted from Iverson [41] and ask participants to rate their agreement with behavioural statements regarding rule violations and speeding, careless driving of others, and drink-driving. Attitudes to risky driving (ATRD) will be evaluated on a five-point Likert scale for each of the 16 ATRD items and summary ATRD scores calculated as the sum of all item scores, after all response data are re-coded so that higher-item scores indicate endorsement of higher-risk behaviours. Summary scores have a possible range of 16–80, with higher scores correlating with more frequent endorsement of higher-risk behaviours [8, 28, 41].

#### Post-trial questionnaire

The post-trial questionnaire includes the same ATRD items as the pre-trial survey and, additionally, it asks participants whether they were involved in any crashes during the 24-week intervention and post-intervention periods that resulted in damage to their own or someone else’s vehicle.

#### Telematics and DrivePoints

The FEEDBACK app will collect the following information from participants whilst they are driving.

1. Mobile phone model and operating system.
2. Latitude and longitude data every 2 s.
3. Vehicle’s speed data every 2 s (measured in metres per second).
4. Duration of the driving trip upon completion.
5. Total distance travelled upon completion of the trip.

This data is processed using industry partner UA’s algorithms to calculate the ‘DrivePoints’ score per participant.

#### Crash data

Police-reported crash data will be accessed through each of the three states’ respective Department of Transportation and will be used to assess the trial’s primary hypothesis. Participants’ licence numbers (collected in the pre-trial questionnaire) will be used to access police-reported crash data. Crash data obtained will include not only binary data, i.e., ‘yes/no’ data on whether a driver was involved in a crash, but also the number of crashes (if the driver was involved in more than one). Data will be obtained for all drivers – both the intervention and control groups – blind to group status.

#### Confidentiality

The survey data gathered through Qualtrics will be exported and stored in MediaFlux and OneDrive, both managed by the University of Melbourne and accessible only to authorised trial staff. Before storing the data, a unique identifier will be generated for each participant, and survey responses that cannot be linked to specific individuals will be stored in MediaFlux and OneDrive. Sensitive and identifiable information such as driver’s license numbers, mobile numbers, and email addresses will be encrypted and stored in MediaFlux. Personal data will be destroyed after each participant’s 28-week trial period. Participants’ driver’s licence numbers will only be shared with their respective state’s Department of Transportation to access information on crash events.

The mobile telematics data collected via the smartphone app will be stored in an AWS S3 bucket and Microsoft SQL Server managed by UA, which has appropriate security measures and privacy in-place for the data stored in their servers. Any survey responses, including demographic details and responses to driver behaviour questions, may be shared in research publications but will not include identifiable information.

### Statistical analysis

A formal detailed statistical analysis plan will be written by the trial biostatistician and published on our research centre’s website while blind to group allocation. The analysis will include all participants according to their randomised allocation.

#### Primary outcome

The primary outcome, whether a young driver had a police-reported crash of any type during the 24-week intervention and post-intervention periods, will be analysed using a log-binomial regression model. Should the log-binomial regression model fail to converge, a Poisson regression model with robust standard errors will be used. The primary hypothesis will be evaluated by obtaining the estimated relative change in the risk of a young driver being involved in a police-reported crash of any type (i.e., risk ratio) for participants in the intervention group compared to the control group, a two-sided 95% confidence interval and a p-value. This model provides valid inference in the presence of missing data if the data are missing completely at random (MCAR). A sensitivity analysis will be conducted using multiple imputation to explore the impact of any deviations from MCAR on the results.

#### Secondary outcomes

The DrivePoints score will be analysed using a constrained longitudinal data analysis (cLDA) model [42]. The response will consist of all DrivePoints scores (scores at baseline, at weekly intervals until 24 weeks post-randomisation) and the model will include factors representing treatment, time (categorical), and treatment-by-time interaction, with the restriction of a common baseline mean across treatment groups.

The total number of police-reported crashes of any type during the 24-week intervention and post-intervention periods will be analysed using a Poisson regression model. Whether a driver has a self-reported crash during the 24-week intervention and post-intervention periods will be analysed using a log-binomial regression model. Driving behaviours (i.e., binary outcomes – speeding, harsh braking, and harsh acceleration at baseline and at weekly intervals from week 4 to week 28) will be analysed using mixed-effects Poisson regression models with robust standard errors. All analysis models will be adjusted for stratification factors, study site and sex.

#### Persistence of treatment effects

To evaluate the persistence of treatment effects after cessation of the treatment and control conditions, analysis models for the DrivePoints score and individual driving behaviours will be used to estimate treatment effects for the post-intervention period (weeks 24 to 28).

#### Effectiveness of personalised incentives

To evaluate the effectiveness of our approach to personalising incentives, we will describe the moderating effect of variation in the presence and strength of financial incentives on estimated treatment effects. This analysis will follow methods from our recent research employing inverse probability of treatment weights-regression adjustment (IPTW-RA) and control function-regression adjustment (CF-RA) to adjust for selection in treatment switching models [28].

## Discussion

Recent research led by the FEEDBACK trial’s investigators highlights that personalised feedback and financial incentives can lead to safer driving behaviours [28, 43]. This trial will build on these findings by assessing the likely impact of personalised feedback and financial incentives on reducing the likelihood of road crashes. The FEEDBACK trial is set to be the largest trial of personalised transport safety feedback and financial incentives using vehicle telematics, globally, and will mark a critical step forward in advancing knowledge around large-scale interventions to reduce road injury.

Moreover, the trial is unique in that it does not rely on participants and researchers remaining in close proximity to ensure that technology is appropriately installed in-vehicle. As such, this trial is not limited to urban areas, but will deliver across three Australian states. The inclusion of rural and regional areas in the study is crucial, particularly as two-thirds of serious injuries and fatal crashes in Australia occur on regional and rural roads [2]. These high rates of regional crashes are not only limited to young drivers, making the findings of the FEEDBACK trial significant for drivers of all ages.

In addition to accounting for the under-representation of rural drivers, the FEEDBACK trial will also generate important knowledge for decision-makers looking to address the over-involvement of young drivers in road trauma. As young drivers are at greatest risk of a crash during the first year of licensed driving [2], it is key that road safety interventions aim to improve driving behaviour among newly licensed drivers. This study will provide evidence of a promising opportunity to achieve these improvements in driving behaviour, and consequently in crash risk, using personalised feedback and incentives.

Finally, the FEEDBACK trial has global significance. The findings from the research will generate knowledge that can be directly translated into transport safety policies associated with young drivers. Further, the increasing market penetration of technologies like mobile telematics highlights the potential for innovative approaches to transport policy around the world. This trial will demonstrate the utility of personalised transport safety feedback delivered using telematics technology and may spur data-informed policy development across the globe.

### Electronic supplementary material

Below is the link to the electronic supplementary material.

Supplementary Material 1

## Data Availability

Not applicable.
